# Flexible Cu Nanostructured Laser-Induced Graphene Electrodes for Highly Sensitive and Non-Invasive Lactate Detection in Saliva

**DOI:** 10.3390/bios16010019

**Published:** 2025-12-25

**Authors:** Anju Joshi, Gymama Slaughter

**Affiliations:** 1 Center for Bioelectronics, Old Dominion University, Norfolk, VA 23508, USA; 2 Department of Electrical and Computer Engineering, Old Dominion University, Norfolk, VA 23508, USA

**Keywords:** laser-induced graphene, copper nanostructures, non-enzymatic lactate sensor, flexible biosensors

## Abstract

A scalable and facile fabrication strategy is presented for developing a flexible, nanostructured, non-enzymatic electrochemical sensor for lactate detection based on copper-modified laser-induced graphene (CuNPs/LIG). A one-step electrodeposition process was employed to uniformly decorate the porous LIG framework with copper nanostructures, offering a cost-effective and reproducible approach for constructing enzyme-free sensing platforms. Scanning electron microscopy and energy-dispersive X-ray spectroscopy confirmed dense Cu nanostructure loading and efficient interfacial integration across the conductive LIG surface. The resulting CuNPs/LIG electrode exhibited excellent electrocatalytic performance, achieving a sensitivity of 8.56 μA µM^−1^ cm^−2^ with a low detection limit of 42.65 μM and a linear response toward lactate concentrations ranging from 100 to 1100 μM in artificial saliva under physiological conditions. The sensor maintained high selectivity in the presence of physiologically relevant interferents. Practical applicability was demonstrated through recovery studies, where recovery rates exceeding 104% showcase the sensor’s analytical reliability in complex biological matrices. Overall, this work establishes a robust, sensitive, and cost-efficient Cu-nanostructured LIG sensing platform, offering strong potential for non-invasive lactate monitoring in real-world biomedical and wearable applications.

## 1. Introduction

Lactate is a key metabolic intermediate generated during glycolysis under both aerobic and anaerobic conditions, making it an essential biomarker for assessing cellular metabolism, tissue oxygenation, and overall physiological status [1]. Elevated lactate levels are closely associated with hypoxia, sepsis, cardiovascular complications, and exercise-induced fatigue, emphasizing the clinical value of accurate and continuous lactate monitoring [2,3]. Conventional analytical techniques provide reliable quantification but are hindered by several limitations, including reliance on centralized laboratories, complex sample preparation, long turnaround times, and the inability to support real-time or point-of-care analysis [4,5,6]. These constraints have accelerated the demand for flexible, miniaturized, and noninvasive electrochemical platforms capable of delivering rapid, sensitive, and field-deployable lactate detection.

Electrochemical biosensors traditionally rely on enzymatic pathways particularly lactate oxidase (LOx) to achieve high selectivity towards lactate [7]. While effective, enzyme-based sensors suffer from intrinsic drawbacks such as poor stability, susceptibility to denaturation, and limited operational lifetime under variable environmental and physiological conditions [8]. To mitigate these issues, LOx immobilization strategies on unconventional substrates, including cotton fabric [9] and laser-induced graphene (LIG) [10], have been explored. Although these sensors exhibit encouraging sensitivity, the instability and degradation of enzymes remain major obstacles to long-term reliability and practical deployment.

Therefore, there has been a pronounced shift toward non-enzymatic electrocatalysts, leveraging nanomaterials with high intrinsic activity, enhanced electron transport properties, and structural robustness. Materials such as metal nanostructures [11], metal oxides [12], noble metal nanoparticles [13], metal–organic frameworks (MOFs) [14], MXenes (Ti_3_C_2_T_x_) [15], and carbon-based nanostructures [16] have demonstrated significant promise for enzyme-free lactate oxidation. Among noble metals, platinum-based composites, such as sepiolite-supported Pt nanoparticles synthesized through chemical reduction, have achieved reliable detection across 10–1100 µM [17]. However, the high cost, limited availability, and fabrication complexity associated with noble metals restrict their scalability. In contrast, transition-metal-based catalysts, particularly nickel derivatives, offer compelling advantages due to their rich redox activity, strong affinity toward lactate, cost-effectiveness, and tunable electrochemical behavior. Ni(OH)_2_ modified indium tin oxide electrodes have demonstrated high sensitivity (0.1325 mA mM^−1^ cm^−2^) and broad detection ranges [18], while NiO-based platforms [19,20], and nickel nanostructures [21] have further improved catalytic efficiency. Additionally, hybrid structures combining nickel with carbon nanomaterials have shown synergistic enhancements in electron transfer and catalytic performance.

Among transition metals, copper-based nanomaterials have emerged as one of the most powerful electrocatalysts for non-enzymatic lactate sensing due to their accessible redox states (Cu^0^, Cu^+^, Cu^2+^, Cu^3+^), low cost, structural diversity, and excellent electrocatalytic capability in both alkaline and neutral media [22]. Several Cu-based systems including mesoporous CuO nanostructures were synthesized via an inverse micelle sol–gel process [23], coprecipitated CuO screen-printed electrodes for sweat analysis [24], and flexible PET electrodes functionalized with MWCNTs and Cu-HHTP MOFs [25] have shown favorable detection ranges and low limits of detection towards lactate. More advanced designs, such as self-supported copper foam structures with 3D open-pore architectures, have delivered ultrahigh sensitivities (800 μA^−1^ cm^−2^ and broad detection windows (1–120 mM) [26], while Cu-Au alloy catalysts have achieved detection limits as low as 5 μM [27].

The existing approaches have been directed towards lactate sensing in serum or sweat primarily, exploring rigid glassy carbon electrodes and drop-cast catalyst inks or composite powders, which suffer from binder dependence, nonuniform porosity, and batch-to-batch variability. Moreover, many non-enzymatic lactate sensors require strong alkaline electrolytes to activate Cu redox chemistry, limiting their applicability in real-world biofluids. Here we explored the use of laser-induced graphene (LIG) as a promising, cost-efficient, and flexible sensing platform, owing to its unique structural and electronic characteristics [28,29,30]. The fabrication process is convenient and straightforward, involving the localized photothermal conversion of polyimide to form a three-dimensional porous graphene network with abundant edge-plane defects, which enhances the electrochemical sensing capabilities [31]. LIG sensors possess excellent electrical conductivity, a large electroactive surface area, and defect-rich active sites that facilitate redox reactions and catalytic processes. However, their electrochemical performance is often limited by structural heterogeneity, high capacitive background currents, and the difficulty in accurately defining the electroactive surface area. Additionally, the defect-rich and porous nature of LIG can lead to surface instability, biofouling, and limited intrinsic selectivity in complex biological media, necessitating surface modification [32].

Saliva is an attractive non-invasive biofluid due to its ease of collection and suitability for frequent monitoring [33]; however, enzyme-free lactate detection in saliva under physiological conditions remains scarcely reported. Animashaun and coworkers reported a flexible (LIG-based non-enzymatic lactate sensor incorporating electrodeposited Au nanoparticles and a PEDOT-based molecularly imprinted polymer (MIP), which achieved excellent sensitivity, a wide dynamic range, and high molecular selectivity through synergistic conductivity enhancement and template-guided recognition [29]. While this approach demonstrates strong analytical performance in artificial saliva, it relies on multi-step fabrication and polymer imprinting strategies that add complexity and may pose challenges for large-scale manufacturability and long-term stability.

Therefore, we present a flexible, LIG sensor modified via a simple, one-step electroreduction of copper nanostructures for non-enzymatic lactate detection in artificial saliva under neutral pH. A systematic investigation of deposition parameters including applied potential and precursor concentration was conducted to optimize Cu nanostructure formation on the conductive graphene architecture. The synergistic integration of electrocatalytically active Cu nanoparticles with the high surface area and excellent charge transport characteristics of LIG enabled sensitive lactate oxidation across 100–1100 µM, achieving a sensitivity of 8.56 μA µM^−1^ cm^−2^ and a low detection limit of 42.65 µM. Scanning electron microscopy imaging revealed dense, uniformly distributed Cu nanoparticles anchored throughout the LIG framework, while elemental mapping confirmed homogeneous deposition. Beyond remarkable sensitivity, the sensor demonstrated strong selectivity against physiologically relevant interferents, highlighting its suitability for complex biological matrices. The ability of CuNPs/LIG to sustain strong electrocatalytic oxidation at pH 7.5 reflects superior material design, as the redox behavior of copper is typically suppressed outside alkaline environments. Thereby demonstrating a proof of concept for flexible lactate monitoring system using an engineered Cu-nanostructured architecture that enables scalable manufacturability, cost-effective fabrication, and stable operation in saliva under near-neutral pH conditions, facilitating direct lactate detection without pH adjustment. This work marks a significant advancement toward realistic, biocompatible, and field-deployable non-enzymatic lactate sensing.

## 2. Experimental

### 2.1. Chemicals and Materials

Polyethylene terephthalate sheets (PET) and polyimide (PI) sheets (thickness = 50 μm) were used for the synthesis of LIG. Potassium ferricyanide [K_3_Fe(CN)_6_] was used as a redox mediator for electrochemical characterization. The synthesis of copper nanostructures on LIG sensor surface involves the use of copper (II) bromide (CuBr_2_), sodium sulphate (Na_2_SO_4_) and sulphuric acid (H_2_SO_4_), which were obtained from Sigma Aldrich, St. Louis, MO, USA. Detailed interference studies were carried out using glucose (Sigma Aldrich, St. Louis, MO, USA), fructose (Acros Scientific, Waltham, MA, USA), uric acid (Sigma Aldrich, St. Louis, MO, USA), dopamine hydrochloride (Alfa Aesar, Haverhill, MA, USA), and Ascorbic Acid (Alfa Aesar, Haverhill, MA, USA). Phosphate-buffered saline (PBS) was prepared using an established PBS recipe from Sigma Aldrich, which involves sodium chloride (NaCl) (Sigma Aldrich, St. Louis, MO, USA), potassium chloride (KCl) (Sigma Aldrich, St. Louis, MO, USA), sodium dihydrogen phosphate (NaH_2_PO_4,_ Sigma Aldrich, St. Louis, MO, USA), and dipotassium hydrogen phosphate (K_2_HPO_4_) (ThermoScientific, Waltham, MA, USA), and was used as a supporting electrolyte during all electrochemical measurements. Deionized water (DI) 18.2 MΩ cm^−1^ was used to prepare all solutions. The artificial saliva was prepared using KH_2_PO_4_ (Sigma Aldrich, St. Louis, MO, USA), K_2_HPO_4_ (ThermoScientific, Waltham, MA, USA), CaCl_2_ (Sigma-Aldrich, St. Louis, MO, USA), NaHCO_3_, and Citric acid from Fisher Scientific (Waltham, MA, USA).

### 2.2. Apparatus

The electrochemical performance of the copper-nanostructured LIG sensor was assessed using a three-electrode configuration on a Metrohm Dropsens µStat-i-MultiX system. In this setup, the CuNPs/LIG served as the working electrode (WE), a platinum (Pt) electrode as the counter electrode (CE), and an Ag/AgCl/3 M NaCl electrode was used as the reference electrode (RE) for all measurements. Morphological and elemental characterization of the electrodeposited CuNPs, and LIG was performed using field-emission scanning electron microscopy (FE-SEM, JSM-IT700HR InTouchScope™, JEOL Ltd., Tokyo, Japan), providing insights into nanoparticle distribution and surface composition.

### 2.3. Fabrication of Laser Induced Graphene Electrodes

A rectangular LIG working electrode (WE) was designed in CorelDraw 2019 and patterned onto polyimide (PI) tape (width: 1.25 cm). The PI tape was carefully laminated onto a flexible PET substrate. The LIG was produced via laser-assisted photothermal carbonization of laminated PI tape using a BOSS LS1416 CO_2_ pulsed laser system operated through LightBurn software (v. 0.909) [34]. The laser parameters including laser speed, maximum power settings, and focal height were systematically optimized to ensure high conductivity, mechanical stability, and reproducibility. The final conditions: 250 mm s^−1^ scan speed, 18% maximum power, 20 mm focal height, 0.1 s laser interval, a single raster pass, and a total irradiation duration of 9 s. This yielded a reproducible 4 mm rectangular WE pattern with a well-defined graphene-based sensor. Following carbonization, a thin layer of nail enamel was applied to precisely define the electroactive area and to insulate the contact pads.

### 2.4. Electrochemical Modification of Cu-Nanostructured LIG Electrodes

For electrode modification, CuNPs were electrochemically deposited onto the LIG surface using a chronoamperometric technique under constant potential. The electrodeposition was performed in an optimized electrolyte bath composed of CuBr_2_, sodium sulphate, and sulfuric acid. To determine the conditions that produced uniform and catalytically active Cu nanostructures, the growth of the CuNPs was optimized under variable deposition potentials and Cu precursor concentrations using a constant potential chronoamperometric technique. CV measurements were recorded from −0.9 to 0.9 V at a scan rate of 50 mV s^−1^ using a standard three-electrode setup.

## 3. Results and Discussion

### 3.1. Electrochemical Synthesis of the Cu Nanostructures on LIG Electrodes

**Figure 1:**
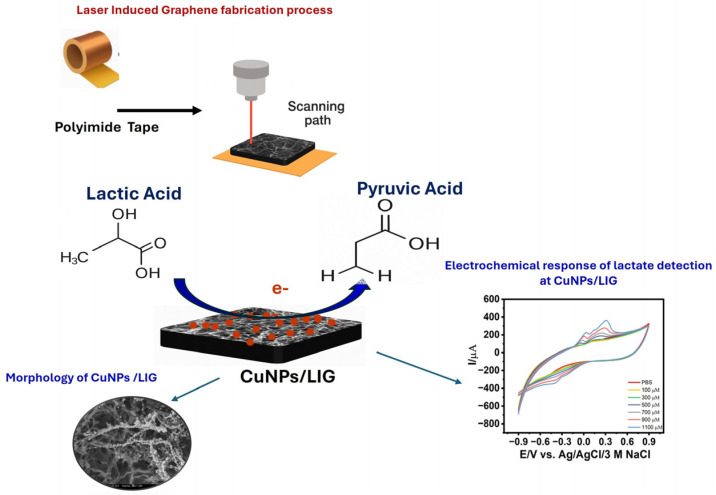
Schematic illustration detailing the steps involved in the fabrication of copper-nanostructured laser-induced graphene (CuNPs/LIG) for non-enzymatic lactate sensing.

The electrocatalytic performance of the CuNPs/LIG electrode toward lactate oxidation was systematically evaluated following controlled electrochemical deposition of Cu nanostructures onto the LIG framework. The electrodeposition parameters, specifically the applied electroreduction potential and Cu precursor concentration were optimized to tailor the surface morphology and catalytic activity of the resulting CuNPs/LIG interface (Figure 1). These parameters are known to strongly influence nucleation kinetics, crystal growth, and the density of catalytically active sites on carbon-based supports. To determine the ideal deposition potential, Cu nanostructures were electrodeposited using CuBr_2_ (10 mM) at varied reduction potentials (−1.1, −1.3, and −1.5 V) for 3 min in an electrolyte containing 10 mM sodium sulfate (Figure S1a, Supporting Information). The corresponding CVs displayed two well-defined anodic peaks at approximately −0.09 V and 0.17 V in the presence of 700 µM lactate, indicating the formation of catalytically active Cu nanostructures at the optimized deposition potential of −1.3 V. In contrast, at a lower deposition potential (−1.1 V), insufficient nucleation was observed, as evidenced by a weaker Cu oxidation peak current (Figure S1a, Supporting Information), leading to reduced electrocatalytic lactate oxidation. Following identification of the optimal deposition potential, the effect of precursor concentration was assessed by varying CuBr_2_ levels while maintaining deposition at −1.3 V (Figure S1b, Supporting Information). Cyclic voltammetry revealed that uniform and highly active Cu nanostructures formed most effectively at 10 mM CuBr_2_, as evidenced by the most prominent Cu oxidation peak and the strongest lactate response. At higher CuBr_2_ concentrations, the CVs displayed broadened and shifted oxidation peaks, indicating mass-transfer–limited growth and reduced electroactive surface area. This behavior is consistent with concentration-dependent mass transport limitations, which at elevated precursor levels may favor aggregation rather than controlled nanoparticle formation [30]. These electrochemical analyses confirm that Cu nanoparticles growth at −1.3 V using 10 mM CuBr_2_ for 3 min yields the most favorable balance of nucleation density, particle size, and catalytic surface exposure and therefore has been considered as optimal. We observed identical chronoamperograms corresponding to the uniform electrochemical modification of the LIG sensors with Cu NPs at the optimized electrodeposition conditions (Figure S2, Supporting Information).

### 3.2. Morphological and Elemental Characterization of CuNPs/LIG Electrodes

**Figure 2:**
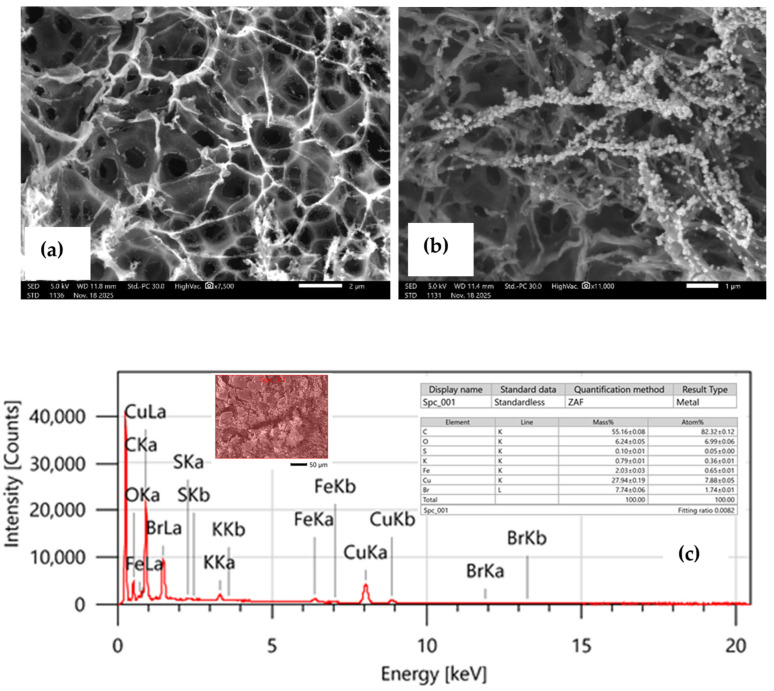
Scanning electron microscopy images of (**a**) LIG; (**b**) CuNPs/LIG; (**c**) Corresponding EDAX analysis for the as-fabricated CuNPs/LIG.

The structural characteristics responsible for the enhanced electrocatalytic performance of the CuNPs/LIG electrodes were investigated via detailed SEM and energy-dispersive X-ray spectroscopy (EDXS) analyses. The SEM micrograph of the pristine laser-induced graphene (Figure 2a) reveals a highly porous, interconnected 3D network formed because of laser-induced photothermal decomposition of polyimide. This process is known to cleave C–O, C–N, and N–C bonds, resulting in turbostratic graphene layers with expanded micro- and mesopores [31,34]. The abundance of large pores and edge-plane defect sites contributes to the high specific surface area of LIG, which enhances electrolyte accessibility and promotes rapid electron-transfer kinetics. Such structural features are essential for improving the conductivity and catalytic performance of LIG-based electrochemical sensors.

Following electrochemical deposition, the morphology of the Cu-modified LIG surface changes markedly, as shown in Figure 2b. The Cu nanostructures appear as densely distributed granular clusters and chain-like assemblies anchored firmly onto the LIG ridges. This configuration suggests an efficient and uniform nucleation-growth process during electroreduction at the optimized potential. The chain-like agglomerates interconnect across the LIG microchannels, forming continuous conductive pathways that facilitate rapid charge transport during lactate oxidation. The intimate contact between Cu nanostructures and the conductive graphene backbone is expected to reduce interfacial resistance, enhance redox cycling, and accelerate reaction kinetics. The granular and clustered morphology is also advantageous because it exposes abundant catalytically active surface sites. Such nanoscale roughness has been widely associated with improved adsorption of lactate molecules and enhanced catalytic turnover on Cu-based sensors. In this context, the observed Cu morphology is consistent with the improved electrocatalytic response recorded under the optimized deposition conditions described earlier.

Elemental composition further supports successful Cu integration onto the LIG framework. EDXS spectra (Figure 2c) confirmed the presence of carbon, oxygen, and copper, with mass percentages of 55.16%, 6.24%, and 27.94%, respectively. The substantial copper content verifies the dense surface loading of Cu nanostructures, while the presence of oxygen is attributed to both residual oxygen functionalities on the LIG surface and native oxide species formed on the Cu nanostructures. Such mixed-valence copper-oxygen species (Cu^0^/Cu^+^/Cu^2+^) are known to contribute synergistically to catalytic activity for lactate oxidation, which further reinforces the suitability of the CuNPs/LIG interface for non-enzymatic sensing applications.

### 3.3. Scan Rate-Dependent Electrochemical Kinetics of Lactate Oxidation at CuNPs/LIG

**Figure 3:**
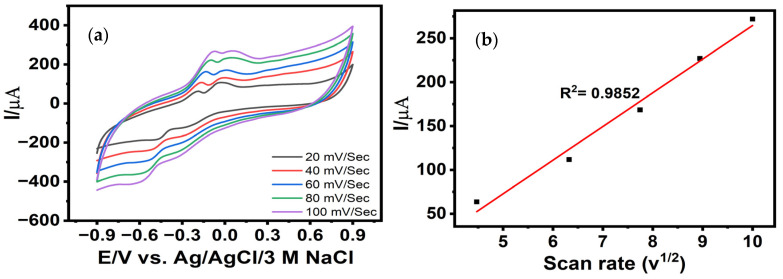
CVs of (**a**) CuNPs/LIG at variable scan rates ranging from 20 to 100 mV s^−1^ and (**b**) Corresponding linear relation between anodic peak current and scan rate v^1/2^.

The effect of scan rate on the electrochemical behavior of the Cu-nanostructured LIG electrode was investigated using CV within the potential window of −0.9 to 0.9 V in the presence of 500 µM lactate. As the scan rate increased from 20 to 100 mV s^−1^, both the anodic and cathodic peak currents exhibited a proportional increase, reflecting the dynamic response of the CuNPs/LIG interface under kinetically accelerated conditions. The anodic peak associated with lactate oxidation, centered at −0.05 V, exhibited a clear linear increase in oxidative current with increasing scan rate (Figure 3a), indicating the rapid electron-transfer capability of the Cu-modified electrode surface. This enhancement in current response can be attributed to the high electrochemically active surface area provided by the uniformly distributed Cu nanostructures embedded within the porous LIG framework. The hierarchical porosity and conductive graphitic domains of the LIG substrate facilitate efficient charge transport, while the Cu nanoparticles serve as catalytic sites, promoting rapid redox transitions during lactate oxidation. The resulting linear dependence between the anodic peak current and scan rate follows the regression:

(1) $$Ip=38.37x-119.38\;(R^{2}=0.9852)$$

indicating strong correlation and excellent electron-transfer performance under varying scan conditions.

Furthermore, the linear relationship between the peak current and the square root of the scan rate (Figure 3b) confirms that the electrooxidation of lactate at the CuNPs/LIG interface is governed by a diffusion-controlled mechanism (Figure S3, Supporting Information) [35]. This behavior is characteristic of systems in which mass transport of the analyte to the electrode surface is the rate-limiting step, rather than surface-limited adsorption processes. The corresponding peak potential shifts positively for anodic peaks and negatively for cathodic peaks, further implying quasi-reversible redox kinetics with increasing scan rate. These shifts are consistent with multistep copper redox transitions (Cu^0^/Cu^+^/Cu^2+^/Cu^3+^) that mediate the electrocatalytic oxidation of lactate, as previously reported for Cu-based catalytic systems [24]. Such behavior highlights the complexity of the electron-transfer processes involved and the suitability of the CuNPs/LIG electrode for sensitive lactate monitoring under dynamic electrochemical conditions.

### 3.4. Electrochemical Characterization and Lactate Sensing Performance of CuNPs/LIG Electrodes

**Figure 4:**
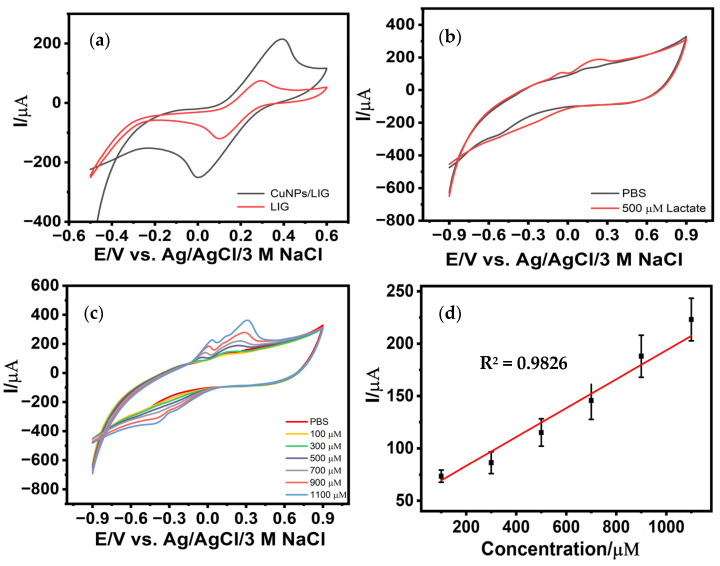
Cyclic voltammograms (CV)of (**a**) CuNPs/LIG and LIG electrodes in 5 mM K_3_Fe(CN)_6_ at a scan rate of 50 mV/s; (**b**) CuNPs/LIG towards electrocatalytic oxidation of lactate; (**c**) CuNPs/LIG under variable concentrations of lactate; and (**d**) Corresponding calibration response between anodic peak current and lactate concentrations (*n* = 3).

The optimized CuNPs/LIG electrodes were first evaluated using the standard redox mediator potassium ferricyanide ([K_3_Fe(CN)_6_]) and subsequently examined for lactate oxidation prior to detailed sensing studies across variable lactate concentrations. The oxidation and reduction peaks for CuNPs/LIG electrodes are characteristic of a quasi-reversible to irreversible redox system with anodic and cathodic peak potentials of approximately 0.392 V and 0.001 V, respectively, and accompanied by significantly elevated peak currents (I_pa_ = 215.42 μA and I_pc_ = −252.82 μA) (Figure 4a). The large peak-to-peak separation, asymmetric peak shapes, and unequal anodic and cathodic peak currents confirm non-ideal reversibility. This behavior is expected for Cu-based surfaces, where multistep Cu/Cu^+^/Cu^2+^ transitions, oxide formation, and surface restructuring limit full regeneration of the initial redox state during the reverse scan [23]. This marked enhancement in redox activity compared to unmodified LIG indicates a substantial improvement in electron-transfer kinetics, arising from the intimate integration of copper nanostructures with the defect-rich, conductive LIG scaffold. The porous graphene network provides a high density of nucleation sites for Cu deposition, while the resulting nanoscale metallic clusters amplify the number of electroactive sites available for redox-mediated processes.

To quantitatively assess this enhancement, the electroactive surface area of the CuNPs/LIG electrode was calculated using the Randles-Sevcik equation from Figure 3a [32]:

(2) $$Ip=2.69*10^{5}*n^{\frac{3}{2}}*A*C*D^{\frac{1}{2}}$$

where *Ip* is the peak current, *n* = 1 for the one-electron process, *C* is the concentration of the redox probe, *D* is the diffusion coefficient, and *ν* is the scan rate [30]. A comparative analysis demonstrated that the electroactive surface area of CuNPs/LIG is approximately 2.75-fold higher than that of the unmodified LIG electrode. This result confirms that the electrodeposition process substantially increases the available catalytic surface by forming a densely interconnected copper nanostructured layer. The porous 3D structure of LIG enhances double-layer capacitance due to its large electroactive surface area and abundant edge-plane defects, resulting in elevated capacitive background currents that can partially overlap with Faradaic signals. Additionally, hierarchical porosity modifies mass transport, enhancing local analyte accumulation while potentially introducing diffusion limitations in thicker Cu-deposited regions. These effects together account for the broadened peaks and quasi-reversible behavior observed in the CVs [36]. The optimized CuNPs/LIG electrodes were subsequently assessed for lactate oxidation (Figure 4b). Two prominent oxidative peaks were observed at approximately −0.05 V and 0.209 V, consistent with characteristic copper redox transitions involved in lactate electrooxidation. These features are attributed to the cycling of Cu^+^/Cu^2+^ species at the electrode surface and reflect the active participation of copper nanostructures in mediating the lactate oxidation pathway. When tested across lactate concentrations ranging from 100 to 1100 µM, the CuNPs/LIG electrodes exhibited a progressive, concentration-dependent increase in both oxidative peaks (Figure 4c). This monotonic response confirms electrocatalytic activity and charge-transfer processes facilitated by the Cu-LIG hybrid architecture. The corresponding calibration plot (Figure 4d) demonstrates a strong linear relationship between the anodic peak current and lactate concentration, expressed by:

(3) $$Ip=0.13x+56.69\;\;\;\;\;\;\;\;\;\;\;\;\;\;\;\;\;\;\;\;\;(R^{2}=0.9826)$$

The slope of the calibration curve and the active surface area were used to calculate the sensor sensitivity (8.56 μA µM^−1^ cm^−2^). The limit of detection (LOD) was determined using 3.3* ϭ/S [37], where *σ* is the standard deviation of the blank (n = 3) and *S* is the calibration slope. The proposed CuNPs/LIG sensor demonstrates a detection limit of 42.65 µM and a linear range of 100–1100 µM, which fully encompass reported physiological salivary lactate concentrations, supporting its suitability for salivary lactate monitoring.

A comparative analysis with previously reported lactate sensors (Table 1) reveals that the CuNPs/LIG electrode offers competitive sensitivity and a favorable detection range, while providing the advantages of a flexible substrate, low-cost fabrication, and non-enzymatic sensing capability at near-neutral pH. Table 1 presents a comparative benchmarking overview of various non-enzymatic lactate sensors reported in the literature, highlighting their differences in electrode materials, electrochemical techniques, and sample matrices. Therefore, direct comparisons of detection limits and sensitivities is interpreted in the context of the sensing mechanism and experimental conditions.

### 3.5. Selectivity, Reusability, and Operational Stability of the CuNPs/LIG Lactate Sensor

Evaluation of sensor selectivity is a critical requirement for determining its suitability for real-sample analysis, particularly in complex biological matrices where multiple electroactive species coexist. To assess interference effects, the electrochemical response of the CuNPs/LIG electrode toward lactate oxidation was evaluated in the presence of common endogenous interferents, such as glucose, fructose, ascorbic acid, dopamine, and uric acid.

**Figure 5:**
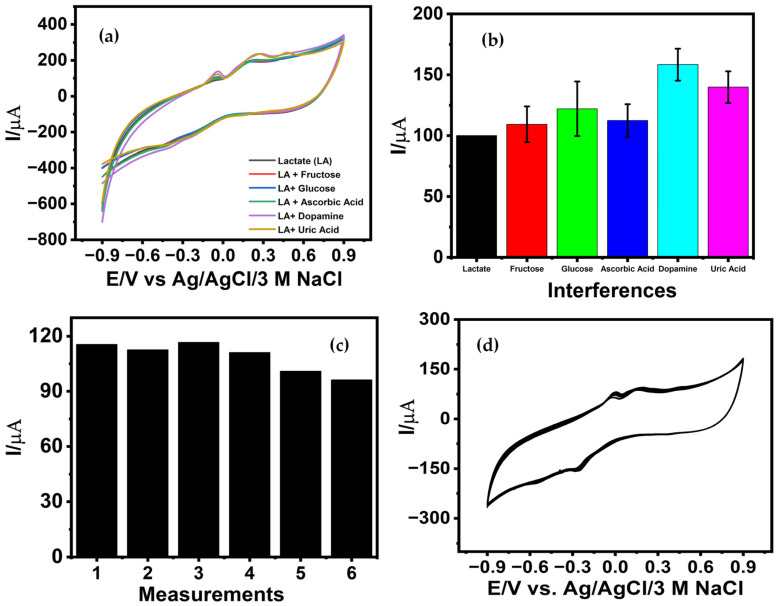
CVs of (**a**) CuNPs/LIG towards lactate (300 µM) and 2-fold co-interferents (ascorbic acid, dopamine, uric acid, fructose, and glucose) n = 3; (**b**) Corresponding bar graph illustrating the influence of interferents toward the electrocatalytic oxidation of lactate; (**c**) Stability of CuNPs/LIG sensor (300 µM lactate); (**d**) Operational stability of CuNPs/LIG under repetitive cycles (300 µM lactate; 20 cycles).

The normal physiological levels of common salivary interferents such as ascorbic acid (40–80 µM), dopamine (0.12 nM), uric acid (100–400 µM), and glucose (30–200 µM), and fructose (1.7–1.9 µM) were considered prior to designing the selectivity study. However, to assess potential cross-reactivity, each interferent was introduced at significantly higher than their physiological concentrations (600 µM each), thereby imposing a more stringent testing condition than real saliva samples. As shown in Figure 5a, all interferents produced an increase in the oxidative peak current; however, the magnitude of this enhancement varied substantially depending on the interferent. Fructose produced only a modest 7.98% increase in current, indicating minimal interference. Glucose and ascorbic acid generated moderate signal increases of 22.29% and 12.5%, respectively, consistent with the partial overlap of their oxidation pathways with copper-mediated redox processes. In contrast, dopamine and uric acid induced more pronounced current elevations of 57.29% and 42.50%, respectively. The significant influence of these species is attributable to their strong electroactivity and their known propensity to undergo facile anodic oxidation on transition-metal-modified electrodes. Moreover, uric acid produced a distinct anodic peak at 0.482 V with a peak current of 244.70 μA, confirming the occurrence of overlapping oxidation processes coexisting with the lactate oxidation response. Additionally, the interference studies were performed using three independently fabricated CuNPs/LIG sensors, and the variability across replicates is represented by error bars in Figure 5b. Although the concentrations of the interfering species are significantly higher than typical salivary levels, the observed current increases of approximately 40–60% for dopamine and uric acid indicate that, if unmitigated, fluctuations in these electroactive species could lead to overestimation of lactate concentration under in vivo conditions. To address this, further improvements in anti-interference performance are required, including optimization of the operating potential window to favor lactate oxidation and the incorporation of thin protective or size-exclusion coatings compatible with flexible substrates. These strategies are expected to suppress interfering signals, enhance selectivity, and improve long-term stability for reliable wearable lactate monitoring [44].

Sensor stability was further evaluated by performing repeated measurements of 300 µM lactate using the same CuNPs/LIG electrode. As shown in Figure 5c and Figure S4, Supporting Information, the first five consecutive measurements produced nearly identical oxidative peak currents, demonstrating minimal signal deviation during early reuse cycles. The decline in peak current observed after the fifth cycle is attributed to progressive surface oxidation and partial passivation of catalytically active copper sites during repeated redox cycling. In addition, adsorption of lactate oxidation intermediates and surface restructuring of the nanostructured electrode can further reduce the number of electrochemically accessible active sites. The observed electrochemical response is consistent with the existing literature [12]. Overall, the sensor retained stable response characteristics for up to five repeated assays, confirming its short-term reusability. Further, the operational stability was assessed subjecting the CuNPs/LIG electrode to 20 consecutive cyclic voltammetric cycles in 300 µM lactate (Figure 5d). The oxidative peak current retained 83.35% of its initial value, indicating strong electrochemical durability and robust adhesion of the electrodeposited Cu nanostructures to the LIG scaffold. This retention reflects a stable catalytic interface capable of sustaining redox cycling without substantial loss of activity.

### 3.6. Practical Evaluation of a CuNPs/LIG Lactate Sensor in Artificial Saliva

The practical applicability of the fabricated CuNPs/LIG sensor was evaluated in artificial saliva prepared following the composition reported by Animashaun et al. [44], with the pH carefully adjusted to 7.5 to closely mimic physiological conditions. For analytical evaluation, the artificial saliva was diluted 1000-fold and spiked with varying concentrations of lactate. Quantification was performed using the standard addition method, and percentage recoveries were calculated to evaluate analytical accuracy. As summarized in Table 2, the sensor demonstrated excellent recovery values ranging from 104.5% to 108%, indicating robust performance in biofluid matrices. Relative standard deviation (R.S.D.) values ranged from 17.5% to 20.83% were observed, which are higher than expected and is attributed to the presence of ionic species and residual organic components that can influence electrode wetting, and nonspecific adsorption, introducing variability in the measured currents.

## 4. Conclusions

Here, we present a facile and cost-effective approach for fabricating Cu nanostructured laser-induced graphene (CuNPs/LIG) electrodes for the detection of lactate. The electrode modification strategy is straightforward, reproducible, and demonstrates a significant electrochemical response toward lactate over a broad detection range, highlighting its high sensitivity and analytical reliability. Importantly, the practical utility of the CuNPs/LIG sensor was validated through lactate detection in artificial saliva, with recovery values ranging from 104% to 108%, demonstrating reliable performance in physiologically relevant matrices. The CuNPs/LIG sensor demonstrates a proof-of-concept study for non-invasive, real-time monitoring of lactate, with potential applications in clinical diagnostics, sports medicine, and personalized health monitoring. The simplicity and versatility of the proposed fabrication approach provide a proof-of-concept flexible platform for lactate detection under the tested conditions. Future development will focus on evaluation in human saliva samples, employing advanced selectivity strategies, and assessment of mechanical robustness under repeated deformation aimed at validating its performance for flexible and wearable applications.

## Figures and Tables

**Table 1:** Comparative performance analysis of the nanostructured electrodes for non-enzymatic lactate detection.

Electrode	Detection Limit (µM)	Detection Range (µM)	Technique	Sensitivity	Sample	Ref.
Ag–PANI/SPE	760	50–4000	CV	0.00176 mA µM ^−1^ cm^−2^	Artificial Saliva	[38]
MoS_2_-AuPt@SPE	0.33	5–3000	SWV	-	Sweat	[39]
Pt@chitosan/ZnTiO_3_NCs/GCE	22.36	300–2400	DPV	0.4529 µA µM^−1^ cm^−2^	Human serum	[40]
NiO(ALD)/CNT-SPCE	67	0–4000; 5000–16,000	CV	138.4 µA mM^−1^ cm^−2^ 39.8 µA mM^−1^ cm^−2^	Saliva	[41]
CoPPc/MWCNTs-COOH	2	10–240	CV	-	Rice Wine samples	[42]
AgNPs/NiO NPs/Polyurethane (PU)	270	600–2200	CA	8.86 nA mM^−1^ mm^−2^	Plasma	[43]
CuNPs/LIG	42.65	100–1100	CV	8.56 μA µM^−1^ cm^−2^	Artificial saliva	**This work**

Ag–PANI/SPE: silver–polyaniline modified screen printed sensor. MoS_2_-AuPt@SPE: gold platinum bimetallic nanoparticles modified molybdenum disulfide nanosheet. Pt@chitosan/ZnTiO3NCs/GCE: Platinum nanoparticle decorated Chitosan/ZnTiO_3_ nanocomposites. NiO(ALD)/CNT-SPCE: Atomic layer deposition of NiO-Coated CNT nanocomposites. CoPPc/MWCNTs-COOH: Cobalt Polyphthalocyanine/Carboxylated Multiwalled Carbon Nanotube Nanocomposites Modified Sensor. AgNPs/NiO NPs/Polyurethane (PU): silver nanoparticles with printed NiO nanoparticles and polyurethane (PU).

**Table 2:** Real sample analysis using CuNPs/LIG for lactate detection in artificial saliva.

Sample	Concentration Added (µM)	Concentration Found (µM)	Recovery (%)	R.S.D (%)
Artificial Saliva	700	756	108.0	17.5
Artificial Saliva	900	941	104.5	20.83

## Data Availability

Data is contained within the article/Supplementary Material.

## Supplementary Materials

The following supporting information can be downloaded at: https://www.mdpi.com/article/10.3390/bios16010019/s1, Figure S1: Cyclic voltammograms (CV) in the presence of lactate (700 µM) using CuNPs/LIG fabricated under (a) variable electroreduction potentials and (b) variable CuBr_2_ concentrations; Figure S2: Chronoamperograms of two identically prepared CuNPs/LIG using 10 mM CuBr_2_ for 3 min at a deposition potential of −1.3 V; Figure S3: Schematic representation of the mechanism associated with the electrocatalytic oxidation of lactate at CuNPs/LIG sensors; Figure S4: Cyclic voltammograms of CuNPs/LIG in the presence of lactate (300 µM).
