# Supplementary material for: Flexible Cu Nanostructured Laser-Induced Graphene Electrodes for Highly Sensitive and Non-Invasive Lactate Detection in Saliva
Source: Biosensors (Basel). 2025 Dec 25;16(1):19. doi: 10.3390/bios16010019 (PMC12839032; doi:10.3390/bios16010019)
Supplement: Supplementary file 1 [file biosensors-16-00019-s001.zip › biosensors-4031668-supplementary.pdf]

biosensors

MDPI

Supplementary Materials

Flexible Cu Nanostructured Laser-Induced Graphene Electrodes for Highly Sensitive and Non-Invasive Lactate Detection in Saliva

Anju Joshi [1] and Gymama Slaughter [1,2,*]

[1] Center for Bioelectronics, Old Dominion University, Norfolk, VA 23508, USA.
[2] Department of Electrical and Computer Engineering, Old Dominion University, Norfolk, VA 23508, USA.
* Correspondence: Author: gslaught@odu.edu

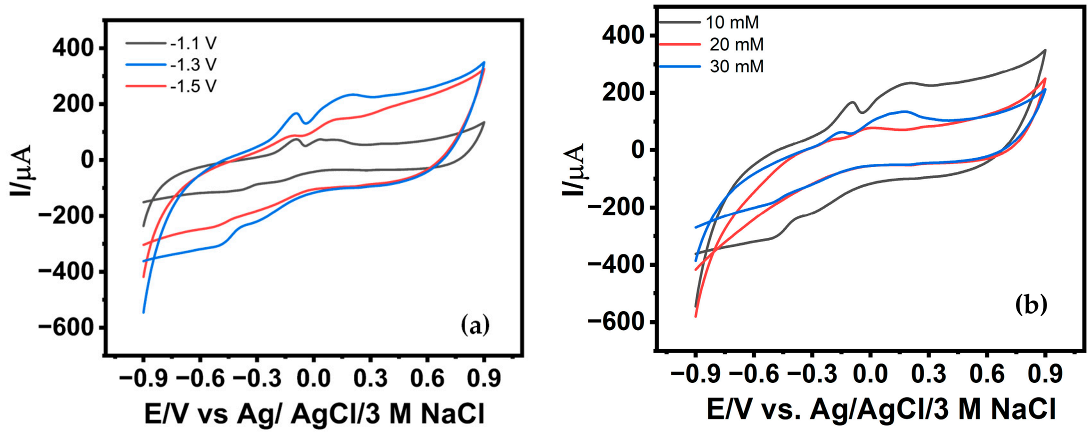

Figure S1. Cyclic voltammograms (CV) in the presence of lactate (700 μM) using CuNPs/LIG fabricated under (**a**) variable electroreduction potentials and (**b**) variable Cu(II)Br concentrations.

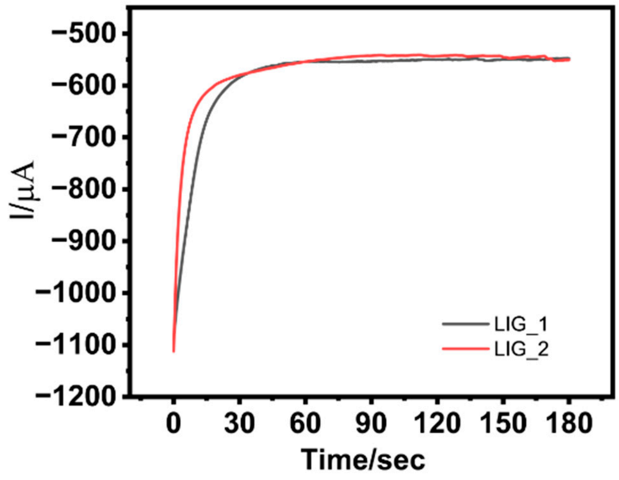

Figure S2. Chronoamperograms of two identically prepared CuNPs/LIG using 10 mM Cu(II)Br for 3 min at a deposition potential of −1.3 V.

https://doi.org/10.3390/bios16010019

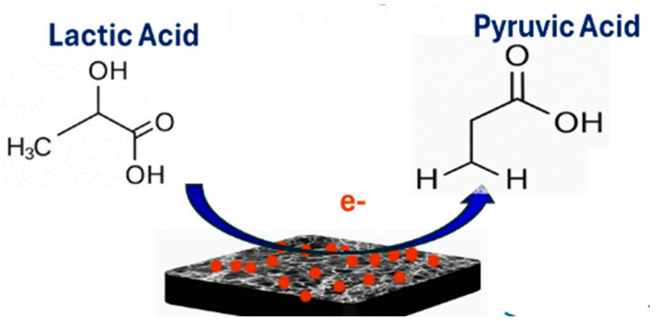

Figure S3. Schematic representation of the mechanism associated with the electrocatalytic oxidation of lactate at CuNPs/LIG sensors.

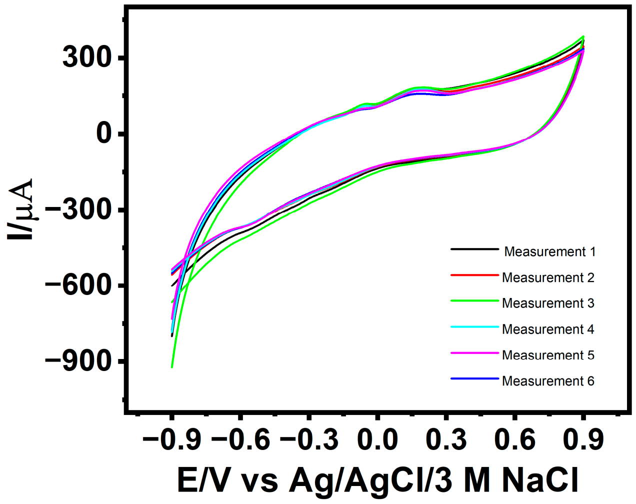

Figure S4. Cyclic voltammograms of CuNPs/LIG in the presence of lactate (300 μM).
